# Navigating a Decade of Integrated Care Research in the International Journal of Integrated Care: How Far Have We Come?

**DOI:** 10.5334/ijic.9086

**Published:** 2025-09-02

**Authors:** Jessica Michgelsen, Nick Zonneveld, Robin Miller, Viktoria Stein, Caroline Longpré, Maripier Jubinville, Nick Goodwin, Mirella Minkman

**Affiliations:** 1 Vilans, Center of Excellence for care and support, The Netherlands; 2 Tilburg University, The Netherlands; 3 University of Birmingham, United Kingdom; 4 Leiden University Medical Centre, The Netherlands; 5 Université du Québec en Outaouais, Campus Saint-Jérôme, Canada; 6 Central Coast Research Institute, Australia

**Keywords:** integrated care, research developments, impact measurement, co-production, people with lived experiences

## Abstract

**Introduction:**

The scope of integrated care has evolved and broadened the past decades from specialist pathways to incorporate a more holistic approach. To identify such trends in evidence and knowledge, we analysed published papers in the International Journal of Integrated Care over a 10-year period.

**Methods:**

From an initial set of 5.075 IJIC papers (2012–2022), 508 articles were selected after excluding several categories such as poster and conference abstracts. As no existing theoretical framework seemed to fit our study aim, we chose to focus specifically on two important areas in integrated care that are known for development; impact measurement and co-production in research.

**Results:**

There was an overall growth of published papers in the journal. The papers predominantly feature contributions from Western regions, including Europe, the Western Pacific and the Americas. Results regarding impact measurement showed no clear overarching pattern over time. Engaging the target population as co-producers in the studies is still low (<5%).

**Conclusions:**

Although the number of papers increased pointing towards more attention for integrated care, we could not identify any significant growth or advancement in the two crucial areas of co-production and impact measurement in integrated care research. These gaps need to be addressed accordingly in both practice and research.

## Introduction

The importance of basing policy and practice decisions on evidence and knowledge is well-established as a mean to build on previous experience and move beyond a reliance solely on personal interpretation and local experience [1]. Working from a substantial knowledge base has the potential to help make sense of the complexity and messiness of health and care, through providing greater clarity of the core elements of interventions, organisational models, the challenges of implementation, and their potential impacts within a system [2]. The creation of ‘evidence’ mainly originates from areas with more developed infrastructures and more funding and publishing opportunities. This can lead to evidence-based decisions being biased towards those approaches and settings which are more able to undertake such studies even though more impactful interventions may be found elsewhere [2]. Research based evidence continues to be central but other forms, notably that of people’s direct experience of accessing health and social care, and the practice knowledge of professionals, are also seen as providing valuable sources of knowledge [3]. There have also been historical tensions between different forms of knowledge, and the respective legitimacy of quantitative and qualitative methods to answer questions of interest [4]. Increasingly the benefits of mixed methods are being recognised as these provide the potential for both measuring impacts at scale and understanding the processes which have resulted in these impacts [5]. All of the above mentioned tensions particularly arise in complex and broad study settings such as integrated care. This field could benefit from a broad scope of knowledge from people with lived experiences, professionals and science.

### Integrated care; complex and broad

Integrated care can be defined as “organizing care and support in a coordinated way, around an individual or group of individuals (and their social network), where boundaries between organizations or sector no longer do not serve as limitations”. It is recognised as a complex endeavour that necessitates commitment and action across multiple levels of the health and care system, involving numerous constituent organisations, professionals, and stakeholder groups [6]. This is further complicated by multiple interpretations of what is meant by integrated care, and its overall purpose, with these ranging from improving the effectiveness and efficiency of health and care services, putting people at the heart of decisions about their treatment and support, and addressing overall health inequalities and support within a population [7]. Integrated care has also changed its scope of interest over the decades, broadening from an initial concern with condition focussed specialist pathways and out-of-hospital discharge processes to incorporate holistic approaches in primary care and social care and supports available within the voluntary and community sector [8].

### A focus on co-production in integrated care research and impact measurement

This has been accompanied by a greater focus on the importance of integrated care in whatever sector being person-centred in its practice and an emphasis on new services and policies being co-produced with people who have lived experience of related services and associated communities [9]. This broadening of scope has highlighted disparities of evidence generation between the different constituencies, new insights in what is seen as ‘good’ evidence, and additional complexity in evaluating impacts through lack of coherent datasets and further complications in attributing outcomes to discrete interventions [10,11]. Furthermore, the multifaceted nature of integrated care not only utilises diverse sources of knowledge and evidence but also encompasses a broad spectrum of disciplines, including public administration, sociology, psychology, organisational science, health economics, epidemiology, and clinical medicine. This makes the use of evidence and other knowledge sources extra complex.

### IJIC as a platform for knowledge exchange

Recognizing the importance of generating relevant evidence, facilitating communication between sectors and countries, and the imperative for interdisciplinary collaboration, the International Journal of Integrated Care (IJIC) was established in the early 2000s. Rooted in a network of professionals spanning various geographical locations and disciplinary backgrounds, IJIC serves as a platform for the dissemination of interdisciplinary research and practical examples of integrated care. Its inception was fuelled by the collective desire to exchange insights, share best practices, and co-create knowledge in the pursuit of more effective, person and community centred approaches to care delivery. Also, the interdisciplinary approach asked for a platform that was not sector or discipline focused. Over the years, IJIC has witnessed the evolution of integrated care from a theoretical concept and political ideology to a practical approach implemented in numerous ways and in various health and care systems around the world. Whilst the scale of evidence published in IJIC has grown steadily over the decades reflecting the increasing interest by governments and international bodies such as the WHO, the extent to which this reflects the breadth and depth of approaches, contexts and stakeholders is not known.

This paper arose from the interest of the editorial board of IJIC in better understanding how the content of the journal has developed over time. In doing so, the journal hoped to identify gaps in evidence and missing methodologies in order to encourage and support authors to meet these opportunities for further evidence and learning. The study also provides an insight into overall developments in the field of integrated care research, as IJIC is a leading international journal focussed specifically on this area of practice and knowledge. The overall aim was to understand the trends in evidence published in IJIC over a 10 year period including characteristics of the papers like the countries of origin and methodologies deployed. We defined two areas of special interest: impact measurement of integrated care and co-production with individuals who have lived experiences. The results offer insights into key journal topics and highlight areas needing more attention in future research and implementation practices. By prioritising co-production, we can ensure that individuals with lived experiences actively contribute to the design, implementation, and evaluation of integrated care interventions. This approach fosters meaningful engagement, enhances the relevance and acceptability of interventions, and ultimately improves the effectiveness and sustainability of integrated care.

## Methods

### Study design and selection criteria

**Figure 1:**
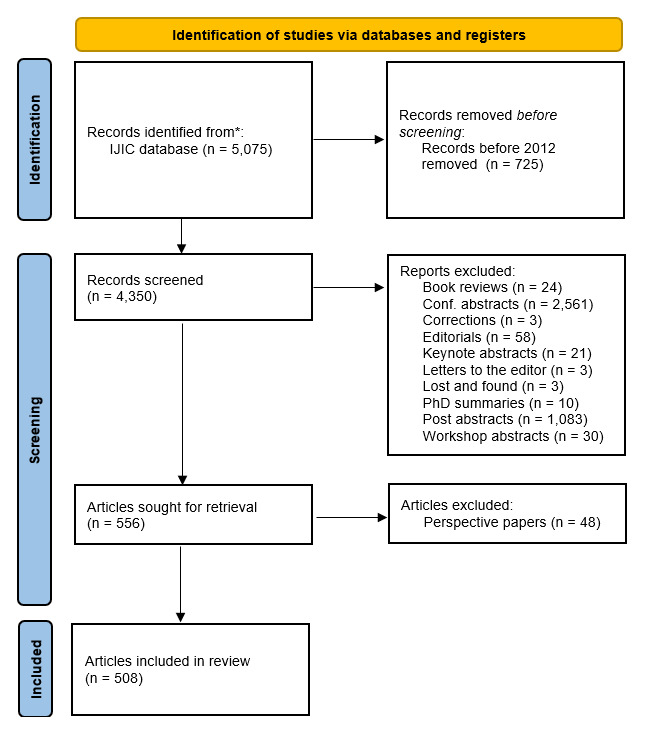
PRISMA flow.

To answer our research aim, a diverse team of researchers conducted a scoping review [12]. The research team consisted of professionals from various countries and functions (research, management, project-based implementation). Step 1 included an article selection procedure with published papers between 2012–2022. We included Research & Theory papers, Policy papers, Perspective papers, Methodology papers and Integrated care cases. The main reason for this was our perception that these paper categories would provide most relevant in-depth insights about the development of integrated care topics over time. We therefore excluded Conference abstracts, Book reviews and PhD thesis summaries from our analysis. In a later stage, we learned that the Perspective papers also did not provide sufficient insights for our study purpose. Hence, the Perspective papers were also excluded from our study. See Figure 1 for more details in a PRISMA flow.

### Data extraction and analysis sheet development

Step 2 involved data extraction and several pilot rounds to develop a data analysis sheet. At a base level, our data analysis sheet included 1) article ID number, 2) initials of the first researcher that analysed the content of the paper, 3) initials of the second researcher that analysed the content of the paper, 4) DOI, 5) title, 6) date of publication, 7) paper category, 8) country/countries in which the study took place, 9) regions in the world, defined by the World Health Organization, 10) methodology, 11) number of citations, 12) number of downloads. To be able to unravel emerging integrated care themes and their development over time, we searched for a theoretical framework/model that could be added to our analysis sheet.

As a first pilot round in search of this theoretical framework, we applied the Knowledge Tree that is built by the International Foundation for Integrated Care (IFIC). This framework includes several key elements such as aligned payment systems to encourage collaboration, strong governance and leadership, and shared values and vision to unite stakeholders. Other elements focus on e.g. monitoring outcomes, digital solutions to improve communication and including people with lived experiences as partners in care. Each researcher involved in this project analysed 10 papers using this Knowledge Tree framework, which means we analysed 70 papers. During a consensus session we shared our reflections. We found the Knowledge Tree insufficient for our study aim as of the large number of broad and different categories. We found that by following this framework, we would not be able to unravel novel and specific enough insights into the development of certain integrated care topics.

In our second pilot round, we incorporated the Development Model for Integrated Care (DMIC) [13] by Minkman into our analysis framework. This model, validated through extensive research, identifies nine critical clusters essential for the successful implementation of integrated care. The first cluster is client-centeredness, which emphasizes the importance of tailoring care to individual needs. Quality care focuses on the standards and effectiveness of care provided. Performance management involves monitoring and improving care delivery. Result-focused learning aims at continuous improvement through feedback and evaluation. Transparent entrepreneurship promotes openness and accountability in care initiatives. Commitment highlights the dedication of all stakeholders to integrated care goals. Roles and tasks clarify responsibilities within the care team. Interprofessional teamwork fosters collaboration across different professional disciplines. Finally, the delivery system addresses the infrastructure and processes necessary for integrated care. Again, seven researchers individually analysed 10 papers each. During a consensus session we concluded that this model also did not provide a sufficient reflection of the content of the papers. In numerous papers, we were able to assign multiple categories to a paper; however, this approach often failed to accurately capture the paper’s central focus. We learned that for our study aim, the conceptual models which have a goal to capture and bring together the multidimensionality of integrated care, are not fit and designed for unravelling (often practise) based studies where these multiple components are intertwined.

As a third pilot round, we decided to abandon the idea of utilising a pre-established conceptual model as a framework for our analyses. Based on our previous experiences and insights during the first rounds, we decided not to analyse the papers on their full content because of their diversity and the intertwinedness of aspects, but to focus on two areas that are known as areas for development. The first focus area relates to the extent to which people with lived experience of health and care are involved in the design and implementation of research and improvement. This both reflects the underpinning values of integrated care and has been highlighted as a gap in research and practice [14,15]. In terms of co-production we follow the definition as used by Marshal & Bamber in 2022 which considers co-production to be a collaborative process involving organisations, practitioners and service users (patients and citizens) to improve community integrated care [15].

The second focus area relates to the time period we have chosen in this paper; a decade of integrated care research. In a timeframe of ten years, one could expect more papers on the impact of integrated care, how to measure it and how to achieve it. Measuring impacts of integrated care is central to decision making over how resources are invested and success is understood. Again this area has been highlighted as being insufficiently studied in both depth and robustness of methods [16]. We chose to primarily define impact based on the quadruple aim categories since it is a widely used framework [17]. We also added space in the analysis sheet for other definitions in case the categories of the quadruple aim were not a proper fit.

The two overarching questions therefore were:

1. To what extent are people with lived experiences co-producers of the integrated care practice and/or research project?
2. To what extent is the impact of integrated care measured?

The third version of our analysis sheet therefore included the elements:

- Who were the study participants?
  - Was it clearly stated they were co-producers of the study?
  - Who were the integrated care service users?
  - Was it clearly stated they were co-producers of the study?
  - Was it clearly stated they were co-producers of the care service?

And the following elements in terms of impact measurement (based on the quadruple aim [17]):

- Has impact been measured?
  - Has impact been measured as service user experience?
  - Has impact been measured as staff experience?
  - Has impact been measured as costs of care?
  - Has impact been measured as health outcomes?
  - Or as something else?
  - What instruments were used?

### First validation session

We presented and discussed the preliminary results of the third version of the analysis sheet during an ICIC workshop in 2022. During this workshop, patient group representatives were present. We presented the progress of our research project, sharing both the search for a suitable analysis framework and the preliminary results. During the group discussions, no one was able to identify a framework that precisely aligned with the scope of our research. We discussed the decision to focus on exploring the impact of integrated care and the degree of co production in integrated care projects; which proved to be solidified by the workshop attendees. By the end of the workshop we took the opportunity to invite fellow researchers to join our project. This resulted in the addition of one researcher to the team.

### Data analysis

In total, 560 papers were analysed by reading the paper and filling in the different categories in the analysis sheet. This was conducted by every member of the project team individually. To ensure the reliability of our individual assessments, 10% of the papers were analysed by a second assessor. An excel database was used to analyse and visualise our findings.

### Second validation session

As a second validation session, we provided a workshop at ICIC 2023. The main goals of this session were to share our findings on impact and co-production, and to reflect on them in smaller groups of researchers from different parts of the world. During this workshop, patient group representatives were present. We additionally used these reflections as input to formulate recommendations for both IJIC and IFIC.

### Keywords analysis

In order to see if we have missed any important topics or trends that changed over time we conducted an additional analysis on keywords. The analysis included a total of 4136 papers published in the International Journal of Integrated Care between 2012 and 2022. The dataset covered a wider range of papers than defined in the selection criteria as the main goal was to validate our earlier made assumptions that co-production and impact would be the most interesting topics to further investigate.

Keywords from the cleaned dataset were extracted and compiled into a comprehensive list using spreadsheet function and techniques. We manually sorted keywords to predefined themes, including care setting, care receivers, care model, impacts, and co-production/co-design based on their semantic relevance. Within each theme, keywords were counted and clusters under each theme were identified using their frequency of occurrence. Temporal trends of keywords were assessed by tracking changes in their frequency over the 10 year period.

## Results

### General descriptives

**Figure 2:**
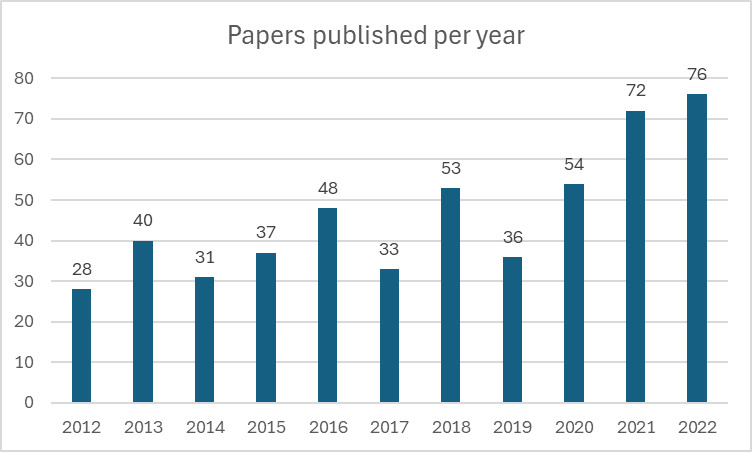
Total number of papers published per year over time in IJIC.

When looking at the total number of papers published in a year in IJIC, there seems to be an overall growth. There was however a decline in papers in the years 2017 and 2019, which can be seen in Figure 2.

**Figure 3:**
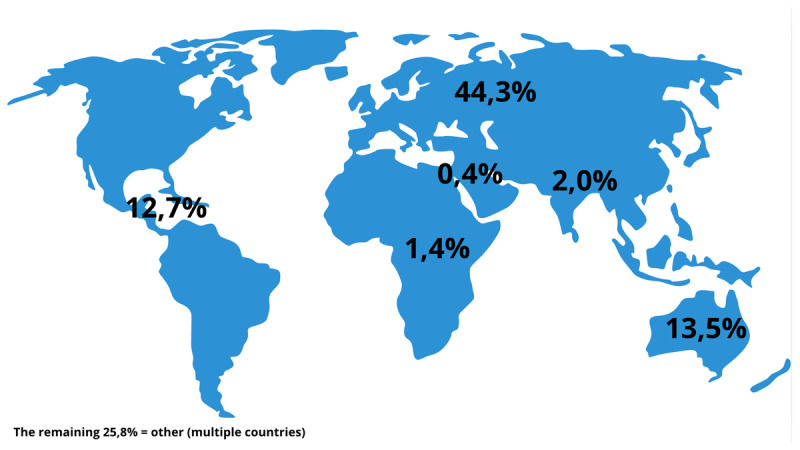
Illustration of where integrated care studies took place (in %).

As shown in Figure 3, the International Journal of Integrated Care (IJIC) predominantly features contributions from Western regions, including Europe (227 papers), the Western Pacific (69 papers, primarily Australia and New Zealand), and the Americas (65 papers, notably the USA and Canada). Conversely, there is limited representation from Southeast Asia (10 papers), Africa (7 papers), and the Eastern Mediterranean (2 papers). Notably, 128 papers lack clear geographical origin, potentially encompassing reviews or studies spanning multiple regions which made it difficult to categorise them into one region.

**Figure 4:**
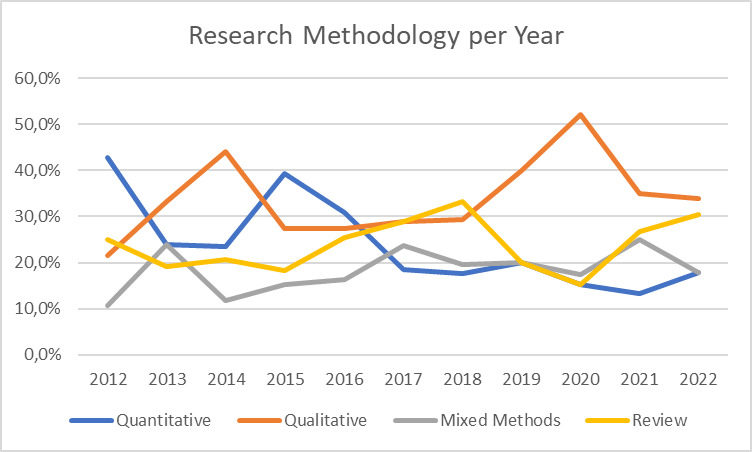
Relative research methodologies published per year (in %).

Figure 4 presents the research methodologies applied in papers published from 2012 to 2022. Quantitative methods show a decline, dropping from 42.9% in 2015 to 17.9% in 2022. Conversely, qualitative methods saw an increase during the COVID-19 period (2019–2020), with approximately 50% of papers using such approaches, but dropped to around 35% after 2021. Reviews and mixed-methods papers exhibit a relatively stable trend over time.

**Figure 5:**
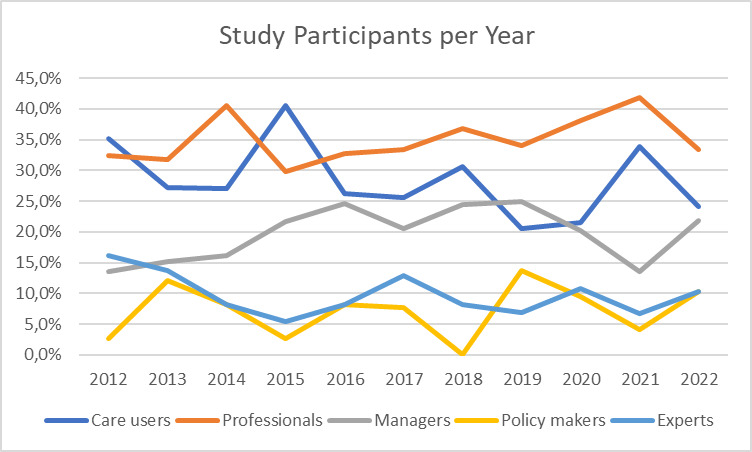
Relative study participant categories published per year (in %).

Figure 5 depicts the primary study participants, with care users and care professionals being most frequently involved. While the percentage of papers studying care professionals shows a slight increase over time, those examining care users exhibit a corresponding decrease. Additionally, managers in care organisations, policy makers, and field experts also feature prominently in studies, with all categories showing fairly consistent trends over time.

### Descriptive results on impact measurement

**Figure 6:**
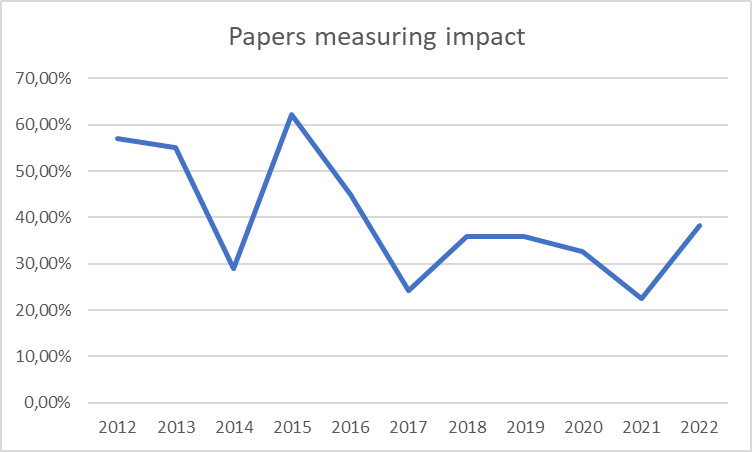
Relative published papers measuring impact per year (in %).

The percentage of papers assessing impact, as depicted in Figure 6, reveal fluctuations over time. While no clear overarching pattern emerges, the data shows periodic shifts, marked by alternating increases and decreases over two to three-year intervals.

### Descriptive results on co-production

**Figure 7:**
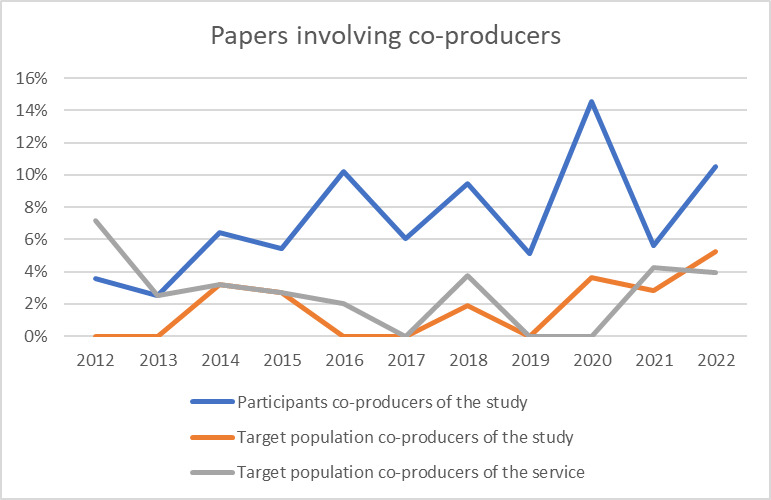
Percentage of published papers per year involving co-producers (in %).

Figure 7 underscores the limited involvement of study participants or the integrated care target population as co-producers in published papers or the development of integrated care services. Notably, only a small fraction of researchers’ papers engage the target population as co-producers in the study, fluctuating between 0% and 4%, or in the development of the integrated care service itself, ranging from 0% to 5%. However, there is a slightly higher level of co-production observed when considering a broader group of participants, such as care delivery professionals, managers, policy makers, and informal caregivers, with percentages ranging from 3% to 15%. This indicates a modest level of engagement beyond the target population, suggesting varying degrees of collaboration and co-production across different stakeholder groups within integrated care studies.

### Keyword clusters by theme

Most of the keywords showed a notable increase in frequency between 2012 and 2017. The keyword “Integration” for example tripled its frequency between 2012 and 2017. This surge can be partly attributed to a concurrent rise in the number of publications during that period reflecting the heightened interest in integrated care models. However, starting in 2019, a sharp decline in the frequency of this keyword was observed. By 2020, the frequency of “Integration” had decreased to just one fifth of its 2012 baseline. These declines align with the possible impact of COVID-19 on publications.

### Care settings

The most common keywords within this theme included “Primary” (Frequency: 442), “community” (Frequency: 312), “Home” (Frequency: 200), “Hospital” (Frequency: 160). These keywords highlight the predominant focus on the corresponding healthcare delivery settings in the literature.

### Care Receivers

Within this theme, the most common keywords were “Patient” (Frequency: 602), “Older” (Frequency: 192) and “Frail” (Frequency: 124). These keyword clusters reflect on the attention given to specific patient populations.

### Care Models

The ‘Care Model’ theme featured “Integrate(tion)” (Frequency: 1944) as the most common keyword, followed by “Collaboration” (Frequency: 254), “Coordination” (Frequency: 172) and “Patient-Centred” (Frequency: 103). These keywords cluster underscore the prevalent research interest in care delivery approaches and innovations. The keyword “integration” is in fact the most common keyword identified.

### Impact

In the “impact” theme, “Outcome” (Frequency: 95) emerged as the most common keyword, followed by “impact & effect” combined (Frequency: 92) and “Cost” (Frequency: 76). The lower frequencies of the keywords compared to keywords in other themes are consistent with the relatively fewer number of studies that assessed impact.

### Co-production

Within the “Co-production” theme, the most common keywords included “Co-design” (Frequency: 60), “Co-production” (Frequency: 35) and “Co-creation” (Frequency: 14). The total number of keywords identified in this theme is relatively low and it is attributed to the proportionally lower number of publications in the dataset that looked into co-production.

## Discussion

This study aimed to understand the trends in evidence and knowledge published in IJIC over a 10 year period. It examined the main characteristics of studies, employed methodologies, roles of people with lived experiences in research and service development, and impact measurement of integrated care. Through this analysis, we sought to guide future research and further development of integrated care services. Before diving into the thematic discussion points, the general reflection of our analysis is that there seems to be a growing interest in integrated care research and policy since the number of papers keeps on increasing.

### Geographical areas

The observed distribution of published papers on integrated care predominantly originating from high-income countries, underscores the pressing need for greater diversity and equitable participation across geographic boundaries within integrated care research. This phenomenon may be attributed to several underlying factors. Firstly, it is plausible that high-income countries tend to have more robust research infrastructures and funding mechanisms, facilitating a higher volume of research projects compared to their low- and middle-income counterparts [18]. In line with this, we see a rise in IJIC publications from China since the past two to three years. Moreover, even when research projects are conducted in low- and middle-income countries, it is often researchers from high-income countries who are the primary authors and publishers, potentially skewing the representation of integrated care practices in research output [19].

There may also be instances where integrated care practices exist in low- and middle-income countries but limited resources and institutional capacities could hinder their documentation and dissemination through research output or have other research traditions. The absence of adequate resources within health and social care systems of certain countries may shift priorities away from integration towards the provision of essential services, posing challenges for the development and implementation of integrated care models. Nevertheless, despite these barriers, integrated care holds significant promise in enhancing service accessibility and efficiency, particularly in resource-constrained settings where individuals stand to benefit from streamlined and coordinated care delivery. Examples of this are HIV cardiometabolic disease outpatient clinic integration, and children’s and women health integration [20]. Additionally, not all health systems are shifting towards integrated care practices which may also explain a lower number of research output from certain areas in the world. Finally, in LMICs, such as most Sub-Saharan African nations, priority is often given to global health and non-Article Processing Charge journals [18]. Consequently, researchers conducting interdisciplinary studies may be more likely to publish their work in global health and public health journals to enhance the visibility of their research than specific integrated care journals such as IJIC. This approach ensures their findings reach a broader and possible other audience and contribute to addressing pressing health challenges in these regions. Thus, while disparities in research output persist, fostering a more inclusive and representative body of knowledge on integrated care remains imperative for addressing global health inequities and advancing the delivery of comprehensive, person and community centred care.

### Underrepresentation of people with lived experiences

One such approach to address inclusion and health inequities is the strategic and systematic participation of people with lived experience throughout the research project, from first conception to publication. At first glance, it was astonishing to see that any direct involvement of patients, caregivers or community members in integrated care research was rare and mostly limited to consultations on results or an invitation to participate in a focus group or survey. For a concept that is per definition person-centred like integrated care, the lack of person involvement bodes ill for a true understanding of what the needs of the service users and the wider community are. There are several possible explanations for this lack of representation, one of them certainly being the lack of funding available to accommodate participatory action research, implementation science or similar approaches, which have at its core the principles of co-design and co-production. Co-produced research is at its core emergent, which means that interventions and outcomes cannot be defined beforehand in such detail as is usually required by clinically driven research funding [21].

Another problem is the lack of knowledge and experience among integrated care researchers to meaningfully involve people with lived experience in their research [22], or put differently, the researchers who work in collaboration with patients, caregivers and community members apparently do not form part of an integrated care research team, since there are plenty of frameworks and tools available to strengthen public and patient involvement (PPI) in research [23]. There is also the possibility that in some cases, researchers believe that with their knowledge of existing evidence and theoretical underpinnings, they are uniquely placed to identify what is worthy of research and their insights of more value than people who directly experience health and care – such power dynamics are present in other aspects of integrated care. Furthermore, it also addresses an important responsibility for society to not spoil research funds for research that does not address needs [24]. It is the responsibility of the publishers of scientific journals to ask for (a statement on) the level of participation (e.g. based on Arnstein 1969) as part of their author guidelines to strengthen the call for participation in research.

Conversely, there was a notable increase in the involvement of professionals and policymakers within integrated care which should be seen as a positive. This trend may reflect lessons learned from numerous pilots and projects over the past decade in which initiatives seen to be successful failed to transition into standard care. A major factor which affects the sustaining and scaling of integrated care innovations is that professionals and managers responsible for implementing changes were not sufficiently informed about the rationale for doing so and the evidence which supported their deployment. Involving those working directly with people and their families, and those responsible for designing and implementing integrated care programmes, within evaluations can help to inform their focus and analysis to ensure that their findings are informative and relevant to those who are in position to take forward their insights.

While the look back in our analysis identified many gaps in PPI approaches in integrated care research, there are some very promising recent developments, which will hopefully reflect on the way integrated care research is reported on in the future: incited by this analysis, the International Journal of Integrated Care embarked on a review of the publication processes, and has recently recruited the first five editors with lived experience on to the editorial board, as well as revising the author guidelines for all articles types to include a statement on the level of participation. The rise of participatory action research and implementation science in integrated care research should also lead to more co-produced research from design to publication. And finally, the move towards more population-based, health and wellbeing focussed models of care in general will strengthen community participation as a necessary element of successful implementation.

### (Un)measurability of integrated care impact

Our study revealed no consistent trends or patterns in the measurement of the impact of integrated care in the IJIC journal over recent years. This suggests that, although the overall number of publications has increased, there has been no corresponding rise in the publication of research specifically focused on measuring the impact, outcomes, or results of integrated care as a concept, method or intervention implemented in real-world practice – at least within the International Journal of Integrated Care.

This observation is noteworthy. It raises the question whether the actual measurement of impact has not been a priority on the research agenda in (health)care management and organisation, whether it has proven too challenging due to methodological complexities, which we also address on a recent editorial [25], or whether such research has indeed been conducted but not widely published within IJIC. Regardless, integrated care continues to be recognised as a key policy paradigm for driving change, as evidenced, for instance, by the national Integrated Care Agreement in the Netherlands. However, time is running out to demonstrate its impact to funders and policymakers, which is crucial for securing its sustainable future. Therefore, whatever the cause may be—whether lack of urgency, methodological difficulties, or limited publication—we strongly encourage more research specifically dedicated to assessing the actual impact of integrated care.

We acknowledge, however, that measuring the impact of integrated care presents significant challenges. In a previous editorial [25], we elaborated on these challenges. We emphasised the importance of: 1) defining what “impact” precisely means for different stakeholders with different values and viewpoints, 2) utilising appropriate tools, for example a Theory of Change methodology, to capture this form of impact while engaging key stakeholders throughout the process, and 3) allowing for a sufficiently extended timeframe to enable a comprehensive assessment of integrated care’s impact on the longer term – as it may take time for impact to become visible and measurable. Even under optimal conditions, a fundamental challenge remains: how can we reconcile the diverse perspectives and values of key stakeholders? [26,27]

This issue leads us to a critical question: How can we effectively evaluate and prioritise these varied viewpoints and values when assessing the impact of an integrated care initiative? As such, one will inevitably need to make deliberate but subjective decisions about which aspects to prioritise when measuring impact. These decisions, in turn, will shape the extent and nature of the impact that can be captured. Another challenge in capturing impact involves limited staff and the pressure this puts on the current workforce since one would want to minimise the added pressure by a research project as much as possible. Connected data systems could be a helpful step forward in this matter. So, capturing the impact of integrated care in its various forms remains crucial and requires a multicomponent character, in a fitting time span, that does not put too much pressure on the workforce.

### Limitations of the study

First, these are insights from one journal and not all the available studies, who might capture more relevant studies. For instance in the case of co-production; other journals may be seen as more applicable in that field of research. Second, we had difficulties in finding or creating a framework that could cover all major topics [13] which in a way forced us to eventually choose some of these topics (i.e. measuring impact and co-production). Third, even after numerous discussions over the course of 2, 5 years, we were not able to find full consensus within this research group on what impact and co-production entail (and therefore also what it does not entail). This could have limited our data analysis since each researcher in this group has analysed parts of the data. Fourth, it proved difficult to find trends over time with the information that we used in our data extraction sheet. Perhaps the trends are more in the details, which we did not capture in this study. More work needs to be done to identify and understand the trends in the body of knowledge on integrated care.

## Conclusions

So to conclude; how far have we come? Because of the large number of studies and multiple aspects within each study we could not identify as many developments over time as one would expect. At least not for the way we include those who receive care services and for how we measure the impact of integrated care. We need to be more strengthened in our research methodologies to capture the impact of integrated care; a set of common approaches to impact measurement would be helpful. We also encourage those who fund research to create opportunities for longer research periods (minimum of 3 years) because both implementation and defining and measuring impact takes a longer time span. As IJIC, we encourage you to publish your research and/or projects that attempted measuring impact but ‘failed’ so that we can all learn from it.

The geographical areas from which the existing body of work on integrated care stems, shows an underrepresentation of practices in low- and middle income areas. What we perceive as integrated care is mainly dominated by researchers from high income countries by which we possibly overlook innovative practices that may emerge in other regions. We therefore strongly encourage different types of research from different areas that somehow relate to integrated care to be submitted in IJIC.

### Next steps for IJIC based on these insights

Firstly, IJIC is currently working on including people with lived experiences in the paper reviewing process. An important element of this is supporting them accordingly so they can fulfil their role to the best of their abilities in a learning environment. Secondly, IJIC will host a special edition on impact measurements in which people are invited to also publish ‘failed’ research projects. Thirdly, the insights of this paper will be shared with the International Foundation for Integrated Care (IFIC) to feed its learning communities and conferences.
