# Correction: Navigating a Decade of Integrated Care Research in the International Journal of Integrated Care: How Far Have We Come?

**DOI:** 10.5334/ijic.11035

**Published:** 2026-04-07

**Authors:** Jessica Michgelsen, Nick Zonneveld, Robin Miller, Viktoria Stein, Caroline Longpré, Maripier Jubinville, Edelweiss Aldasoro, Nick Goodwin, Mirella Minkman

**Affiliations:** 1 Vilans, Center of Excellence for care and support, The Netherlands; 2 Tilburg University, The Netherlands; 3 University of Birmingham, United Kingdom; 4 Leiden University Medical Centre, The Netherlands; 5 Universitédu Québec en Outaouais, Campus Saint-Jérôme, Canada; 6 International Foundation for Integrated Care, Spain; 7 Central Coast Research Institute, Australia

**Keywords:** integrated care, research developments, impact measurement, co-production, people with lived experiences

## Abstract

This article details a correction to: Michgelsen J, Zonneveld N, Miller R, Stein V, Longpré C, Jubinville M, et al. Navigating a Decade of Integrated Care Research in the International Journal of Integrated Care: How Far Have We Come? *International Journal of Integrated Care*. 2025;25(3):25. DOI: https://doi.org/10.5334/ijic.9086

## Correction

The article “Navigating a Decade of Integrated Care Research in the International Journal of Integrated Care: How Far Have We Come?” [1] was published with an incomplete author list. Edelweiss Aldasoro contributed to the analyses in this paper but was not listed as co-author. Her contribution was overlooked due to the corresponding author’s change in affiliation and resulting loss of access to relevant communications. The author list has been corrected in the original article.
